# Frequency of remnants of sealants left behind in pits and fissures of occlusal surfaces after 2 and 3 years

**DOI:** 10.1007/s00784-016-1766-7

**Published:** 2016-03-10

**Authors:** Xuan Hu, WeiWei Zhang, MingWen Fan, Jan Mulder, Jo E. Frencken

**Affiliations:** 1 The State Key Laboratory Breeding Base of Basic Science of Stomatology (Hubei-MOST) & Key Laboratory of Oral Biomedicine Ministry of Education (KLOBM), School and Hospital of Stomatology, Wuhan University, Wuhan, 430079 People’s Republic of China; 2 Department of Oral Function and Prosthetic Dentistry, College of Dental Sciences, Radboud University Medical Centre, P.O. Box 9101, 6500 HB Nijmegen, The Netherlands

**Keywords:** Sealant retention, Glass-ionomer cement, Glass-carbomer, Resin composite, Pits and fissures

## Abstract

**Objectives:**

The null-hypothesis tested was that there was no difference in the frequency of remnants of high-viscosity glass-ionomer sealants left behind in pits and fissures of occlusal surfaces of first permanent molars and that of resin composite and glass-carbomer sealants.

**Materials and methods:**

Based on the results of a sealant trial, a sample of sealed teeth from which the material had apparently completely disappeared from at least one of the three sections into which the occlusal surface was divided, assessed through visible clinical examination, was also assessed from images of colour photographs and Scanning Electron Microscopy (SEM) as the reference image. The sample size consisted of 112 and 120 teeth from 59 and 98 children at evaluation years 2 and 3 respectively. Two examiners performed the assessments. Fisher’s Exact Test was applied to test for the differences between the dependent variable and the sealant groups.

**Results:**

The remnants of sealant material left in the deeper parts of pits and fissures were assessed from colour photograph and SEM images in five sections at year 2 and in eight sections at year 3. The assessment found no sealant group effect.

**Conclusion:**

The frequency of remnants of glass-ionomer sealant in pits and fissures of occlusal surfaces in first permanent molars is not higher than the frequency of glass-carbomer and resin sealants after 2 and 3 years.

**Clinical relevance:**

Contrary to the current assumption, there appears to be no significant difference in the frequency of remnants left behind in pits and fissures between glass-ionomer and resin sealants.

## Introduction

A sealant is placed with the intention to cover a pits and fissures system and for it to remain there for a long time. It will enhance the probability of preventing the onset and progression of a carious lesion. However, sealants deteriorate over time. The deterioration process often results in small or large parts of the material having disappeared, re-exposing the enamel surface to the oral environment. The rate of sealant disappearance varies across brands of sealants but is usually higher among glass-ionomer- than resin-based materials [1]. Among the glass-ionomers, retention of the high-viscosity type (HVGICs), particularly when applied under finger pressure as part of the Atraumatic Restorative Treatment (ART) approach, is on average higher than for the medium-viscosity type [2]. Encapsulated high-viscosity glass-ionomer cements show a higher rate of retention than the hand-mixed version [3].

Despite the early exposure of enamel to the oral environment, the failure rate, expressed in the development of a cavitated dentine carious lesion, is not higher for HVGICs than for resin composite sealants [4–6]. This phenomenon led Frencken and Holmgren [7] to state that retention should be considered only a surrogate endpoint of sealant effectiveness, the true endpoint being the prevention of the occurrence of a cavitated dentine carious lesion. This statement has been investigated meanwhile. A systematic review showed that loss of sealant retention appears to be an invalid predictor for clinical outcome [8] and should not be considered even a surrogate endpoint [9]. The last-mentioned study also showed that the ‘risk of loss of complete retention of sealant materials was associated with the risk of caries occurrence for resin but not for GIC-based sealants’. In other words, a high level of sealant retention is more important when a resin- rather than a glass-ionomer-based sealant is used for maintaining a healthy pits and fissure system.

What could be the reason(s) for the unexpected outcome of the systematic review? It is generally accepted that sealants should be placed in high-caries risk pits and fissures of children with a high-caries risk at mouth level. This treatment should be supported by proper oral hygiene measures performed by the child and/or parents. These oral hygiene measures should move the child from a high- to a low-caries-risk status at mouth level over time. One reason for the difference between GIC-based and resin-based sealants may be the effect of child and parent education regarding proper plaque control in the studies that have been included in the systematic review, but this seems to be very unlikely. A more plausible reason may be related to the characteristics of the two types of sealant material. Glass-ionomer fractures cohesively, leaving behind remnants to the enamel. If this happens in the deeper parts of the pits and fissures system, these systems then become less deep, which increases the chance of the extensive removal of plaque with brush and toothpaste from otherwise inaccessible pits and fissures.

As very few studies that investigate the frequency of remnants of glass-ionomer- and resin-based sealants left behind in pits and fissures have been carried out, a secondary investigation into this phenomenon was performed as part of a sealant trial in China, in which glass-carbomer sealants had also been investigated [10, 11]. The null-hypothesis tested was that there is no difference in the frequency of remnants of high-viscosity glass-ionomer sealants left behind in pits and fissures of first permanent molars of resin composite and glass-carbomer sealants.

## Material and methods

### Background of the sealant trial

In 2008, a sealant trial started in Wuhan, China, covering 407 high-caries-risk children, aged 7.0 to 9.1 years, from five primary schools [10, 11]. A total of 1344 first permanent molars with a deep and/or intermediate pit or fissure system in the occlusal surface were sealed with a resin, a glass-carbomer or a conventional high-viscosity glass-ionomer that had been auto- and thermo-cured with a high-intensity lamp applied according to the ART concept (Table 1). Children in this randomised controlled clinical trial that used a parallel group design were allocated to the four sealant groups, using a list obtained after block randomisation (12 children per block for three operators with each block randomised differently using computer-generated numbers (operators) and letters (sealant groups)) prepared by a statistician who was not involved in the data analyses. Three dentists who were assisted by a chairside assistant each placed the sealants. Before the start of the study, the teams had been trained for 4 weeks in placing sealants, understanding the study set-up and recording data.

**Table 1:** Trade name, manufacturers, composition and batch numbers of materials used to seal pits and fissures in the present study

Trade name	Manufacturer	Composition	Batch number
Ketac Molar Easymix	3MESPE	Glass-ionomer	355998/299174
Glass-carbomer	First Scientific	Glass-carbomer	1602005/8610273
	Dental		
Clinpro	3MESPE	Resin composite	20070416/7JF

During the 2 months intervention period, the children were instructed to brush their teeth before treatment, and all the included first permanent molars were sealed at the school compounds using portable equipment and artificial light. For most of the children, the sealant placement was the first procedure performed in their mouth by a dentist.

Isolation for all types of sealants was obtained through the use of cotton wool rolls. Before placing a sealant in groups 1–3, plaque and debris were removed from pits and fissures using an explorer and a wet cotton wool pellet (ART procedure).

*Group 1 Glass-ionomer*: Ketac Molar Easymix® (3MESPE, Seefeld, Germany): positive control. Sealant application followed the ART sealant procedure. After cleaning the occlusal surface, pits and fissures were dried with dry cotton wool pellets, conditioned with a moist pellet dipped in the glass-ionomer liquid for 10 s, then washed twice with wet cotton wool pellets and dried with dry ones. Glass-ionomer powder and liquid were mixed within 30 s, applied to the surface with an applier/carver ART instrument (Henry Schein, Chicago, USA) and firmly pressed into place for 5–10 s by a petroleum jelly coated index finger (press-finger technique). After bite check, excess material and the petroleum jelly-coated top layer were removed using the applier/carver ART instrument. The smooth, curved angle of the ART applier instrument was used for burnishing the surface which was finally, covered with a new layer of petroleum jelly. Children were advised not to eat or bite for at least 1 h.

*Group 2 Glass-ionomer light-cured*: Ketac Molar Easymix® plus LED high-intensity curing light, Elipar™ Freelight 2, (3MESPE, Seefeld, Germany), producing 850 mW/cm^2^: test group. The sealant application described for group 1 was followed, except that the sealant was cured for 60 s after burnishing.

*Group 3 Glass-carbomer*: Glass Carbomer® (First Scientific Dental, Elmshorn, Germany): test group. Surface cleaning was done under cotton wool isolation as described for group 1, followed by applying Glass-Carbomer Tooth Cleaner (First Scientific Dental, Elmshorn, Germany) over the tooth surface for 20 s and washing and drying of the surface with two wet and dry cotton pellets, respectively. The Glass Carbomer® capsule was mixed for 15 s in a Rotomix™ (3MESPE, Seefeld, Germany), extruded onto the tooth surface, spread into a thin film, covered with Glass Garbomer Surface Gloss (First Scientific Dental, Elmshorn, Germany) and held under finger pressure for 5–10 s. After bite adjustment, the material was light-cured for 75 s with the same LED lamp as used in group 2.

*Group 4 Composite resin*: Clinpro® (3MESPE, Minneapolis, USA): negative control. The pits and fissures were cleaned with a rotating brush: Prophy Angle (3MESPE, Wuhan, China) and a no. 6 explorer, acid-etched with Scotchbond™ etchant (3MESPE, St. Pauls, Minneapolis, USA) for 20 s, rinsed and dried using a portable suction machine. The sealant material was placed in the pits and fissures, manipulated with an explorer to free potential air bubbles and cured for 20 s with the LED curing light 1 mm above the surface. Carbon paper and rotary instruments were used in bite adjustment. The trial was approved by the Research Ethics Committee of Wuhan University, reference number 200704 and was registered at the Dutch Trial Registration Centre, reference number 1441.

### Study set-up

An additional aim of the sealant trial was to assess whether or not sealant material had been left behind in pits or fissures that had received a score of 4, 5 or 6 (Table 2) at the 2- and 3-year clinical examination and whether the outcome was different for each of the sealant materials used. To fulfil the requirements of this secondary study, within the budget of the study, a convenience sample was drawn by selecting every fifth child at year 2 and every seventh child at year 3, from a list of children having sealed teeth with a score of 4, 5 or 6 in, at least, one section of a maxillary or a mandibular first molar. Codes 5 and 6 and sealant material were stratification factors and counted for 25 % of the population of sampled teeth. The assessment was performed from colour photographs and scanning electron microscopy (SEM) images.

**Table 2:** Evaluation criteria for assessing sealant retention through the visual clinical examination

Code	Description
	Tooth is cleaned and dried with a cotton stick
1	Pit and fissures completely covered with material
2	Pit and fissures partly visible. Sharp fracture edge (creating plaque retention site)
3	Pit and fissures partly visible. Crumbled fracture edge (not creating plaque retention site)
4	Pit and fissures totally visible
	If code 4 is recorded then pits and fissures are re-observed after the tooth surface is blown dry with compressed air. Code 4 can be then replaced by code 5 or 6
5	Pit and fissures totally covered with remnants
6	Pit and fissures partly covered with remnants
7	Other treatment performed (new sealant or a restoration)
9	Unable to diagnose

### Preparation of Scanning Electron Microscopy images

The first author took an impression of the sampled teeth following the manufacturer’s instructions. The light body polyvinyl siloxane (PVS) impression material Express (3MESPE, Seefeld, Germany) was syringed onto the dried occlusal surface while an assistant hand-mixed a putty PVS (Express, 3MESPE, Seefeld, Germany) and placed it in a partial dental impression tray (Qingpu Nikang Dental Instrument Manufactory, Shanghai, China). The tray was positioned over the syringed sealed tooth and, after its removal, was rinsed under tap water. Thereafter, the impressions were taken to the Key Laboratory building of the School of Stomatology, Wuhan, and were cast with epoxy resin (Epofix, Struers A/S, Ballerup, Denmark) according to the manufacturer’s instructions.

The epoxy resin tooth specimens were mounted on aluminium stubs, using double-sided adhesive tape, in such a way that the area to be studied faced upwards. After air-drying, the mounted surfaces were coated with a 25-nm-thick layer of pure gold using an ion sputtering unit (Sputter Coater 005, BAL-TEC Inc., Balzers, Liechtenstein). The stubs were then placed in the vacuum chamber of the Scanning Electron Microscope (Quanta200, Philips-FEI Co., Netherlands) of the State Key Laboratory of Geological Processes & Mineral Resources, China University of Geosciences in Wuhan. The accelerating voltage (20 kV) and the aperture were adjusted to suit the specimen for optimisation of the quality of the SEM image. The tooth surfaces of the specimens were observed, adjusted and scanned using different magnification (×10, ×25 and ×50), photographed and stored as tagged image file format (TIFF) files.

### Preparation of colour photographs

An intra-oral photograph using digital camera EOS 400D (Canon, Tokyo, Japan) with ring-flash and macrolens of 100 mm, f/2.8 features of the selected teeth, was taken by a trained photographer with assistance from one of the authors. Photographs of mandibular molars were taken, with the children seated on a chair, while the maxillary molars were photographed with the children lying on a table. Each photograph was judged for acceptability and quality and if not acceptable the photograph was retaken. The photographs were cropped to show only the sealed tooth and then randomly ordered to ensure that the identity of the material was not known to the evaluator.

### Evaluation using visual clinical examination

Prior to the examination, children brushed their teeth at a sink on the school compound. Visual clinical examination was performed by two calibrated and experienced independent evaluators at 0.5, 1 and 2 years and by two calibrated and experienced but different colleagues at evaluation points 3 and 4 years. The evaluators used the criteria presented in Table 2. The first molars were divided arbitrarily into three sections (mesial-central-distal) in mandibular teeth and into two sections (central and distal) in maxillary teeth. An intra-oral light with a disposable mirror attached (Mirrorlight, Kudos, Hong Kong) was used to illuminate the examination site. Any remaining visible plaque or debris was removed with the aid of an explorer or cotton stick. The sealed tooth surfaces were dried with a cotton stick. Trained recorders assisted the evaluators. If sealant material was judged to have disappeared completely from a section or from the total tooth surface, the re-exposed pit and fissures were dried with an air syringe and judged again to see if remnants were visible in the deeper parts of the pit and fissure system (Table 2).

The kappa coefficient values for the inter-examiner consistency related to the visual clinical assessment of sealant retention of the sealant trial were 0.62 and 0.97 at the 0.5- to 2-year, and 3- and 4-year evaluation points, respectively [11].

### Evaluation using colour pictures and scanning electron microscopy images

**Fig. 1:**
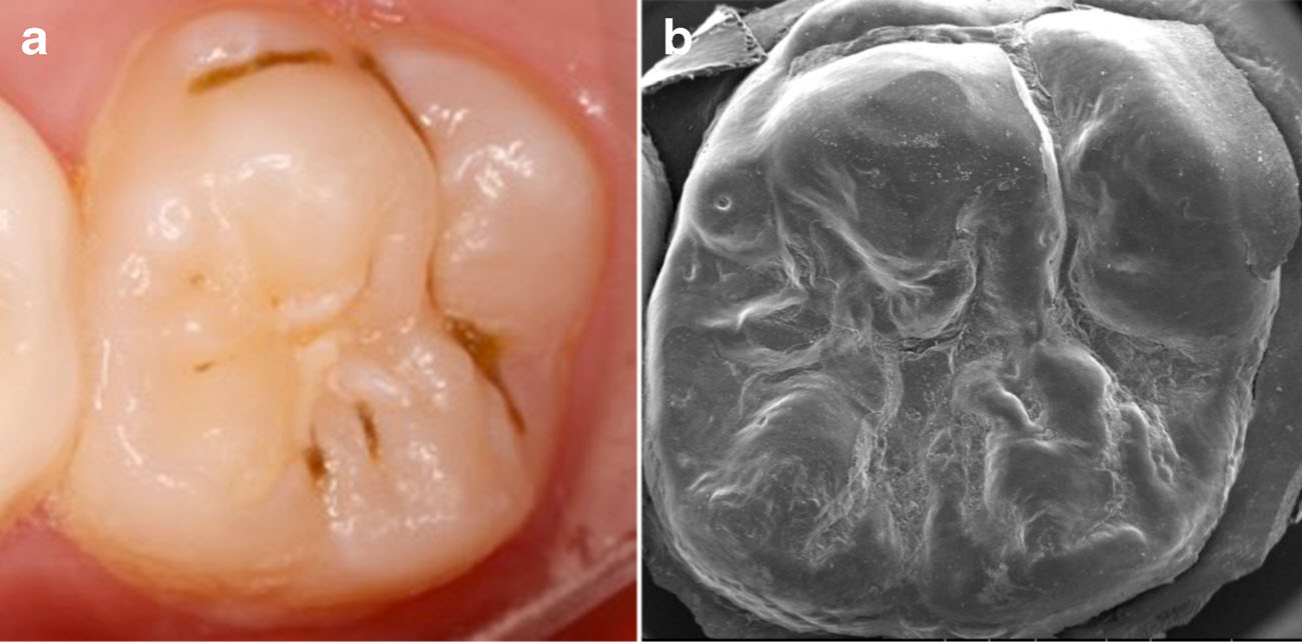
Tooth 26 evaluated after 3 years; clinical picture (**a**) and SEM image at 10× magnification (**b**). The central section on the clinical picture contains a sealant that partially covers the fissures. Whether remnants are present in the re-exposed part of the central section and in the distal section is difficult to see. Assessing these sites from the SEM image is also difficult because of the various different structures visible that may show arrested enamel carious lesions on the clinical picture

Some SEM images (2 % of sections) were poor due to the presence of unidentifiable bodies and structures in pits and fissures while other SEM images (3 % of sections) were difficult to interpret (Fig. 1). These facts made us deviate from the study protocol by not assessing sealant retention from SEM images only, as done in the past [12–14], but relying on colour pictures principally. This decision was possible because the assessment of sealant retention from colour pictures showed high sensitivity and specificity values [15]. The SEM images were used in conjunction with the colour pictures, resulting in a combined assessment of sealant retention.

The level of sealant retention in the occlusal surfaces was assessed using the criteria described in Table 2. At the 2- and 3-year evaluation points, two calibrated evaluators, who were not involved in any of the previous examinations, assessed the absence or presence of sealant material on all sections of the molars from colour photograph images first, followed by the SEM images at ×10 magnification. The colour photographs were placed in an MS Word document, three on one page. The colour photograph and SEM images were viewed on a 12.1-in. monitor with high resolution (1024 by 768 pixels) using ‘Adobe Photoshop CS version 4.0’ software (Adobe Systems Inc., San Jose, USA). Concordance of opinion was reached by discussion.

The kappa coefficient values for the inter-examiner consistency for the assessment of sealant retention on mesial, central and distal sections from colour photographs and SEM images combined were 1.0, 0.94 and 0.79, respectively.

### Statistical analyses

The differences between the four sealant groups for retention code 4 assessed through visible clinical examination and from colour photograph and SEM images were tested with the Fisher’s Exact Test. Because of the very low numbers of teeth scored retention codes 1 and 2, these scores were combined in the analyses. A significant difference was set at *p* = 0.05.

## Results

### Background information

The number of evaluated teeth with sealed occlusal surfaces in the 380 and 371 remaining trial children after 2 and 3 years were 1254 and 1138, respectively. The sampling procedure resulted in 112 and 120 sampled teeth from 59 and 98 children at years 2 and 3, respectively. The number of tooth sections was 271 and 296 at years 2 and 3, respectively.

### Retention code 4 from visible clinical examination at years 2 and 3

The results of the association between clinical retention code 4 and retention codes obtained from assessing images of colour picture and SEM for the three sections of the occlusal surface by sealant group at evaluation years 2 and 3 are presented in Tables 3 and 4, respectively. No code 5 was scored from colour photographs and SEM images and only five times code 6, divided over the four sealant groups at evaluation year 2. For that year. an overall statistically significant difference between the four sealant groups was found (*p* < 0.0001). Comparison of each group with the others showed that only the resin group differed statistically significantly from the others. A test of the resin group against the combined three other groups was also statistically significantly different (*p* < 0.0001). The difference was related to the outcome that 50 % of clinical retention codes 4 had either been scored retention code 1 (27.2 %) or retention code 2 (22.8 %) from assessing colour photographs and SEM images.

**Table 3:** Clinical retention code 4 against retention scores assessed from images of colour picture and SEM (code) by sealant group for the three sections (mesial-central-distal) of the occlusal surface at evaluation year 2

	HVGIC/ART				HVGIC/ART thermo-cured				Glass-carbomer				Resin composite			
Assessment criterium	Mesial	Central	Distal	Total	Mesial	Central	Distal	Total	Mesial	Central	Distal	Total	Mesial	Central	Distal	Total
Code 1	0	1	0	1	1	0	0	1	0	0	0	0	5	4	3	12
Code 2	2	4	4	10	0	3	2	5	1	2	1	4	1	3	6	10
Code 4	12	7	18	37	9	13	15	37	13	20	25	58	7	5	9	21
Code 6	0	0	1	1	0	1	1	2	0	0	1	1	0	0	1	1
Total				49				45				63				44

*HVGIC/ART* high-viscosity glass-ionomer cement used with the Atraumatic Restorative Treatment procedure

**Table 4:** Clinical retention code 4 against retention scores assessed from images of colour picture and SEM (code) by sealant group for the three sections (mesial-central-distal) of the occlusal surface at evaluation year 3

Assessment criterium	HVGIC/ART				HVGIC/ART thermo-cured				Glass-carbomer				Resin composite			
	Mesial	Central	Distal	Total	Mesial	Central	Distal	Total	Mesial	Central	Distal	Total	Mesial	Central	Distal	Total
Code 1	1	0	0	1	0	0	0	0	0	0	0	0	0	1	0	1
Code 2	0	0	0	0	0	0	2	2	1	2	0	3	3	0	1	4
Code 4	11	10	17	38	9	13	13	35	17	21	24	62	5	9	15	29
Code 6	0	1	3	4	0	1	1	2	0	1	1	2	0	0	0	0
Total				43				39				67				34

*HVGIC/ART* high-viscosity glass-ionomer cement used with the Atraumatic Restorative Treatment procedure

At evaluation year 3, no retention code 5 and eight times code 6 (six times for the two HVGIC/ART groups together and none for the resin group) were observed when the assessment was performed from colour photographs and SEM images. No effect was observed of the association between retention code 4 from clinical investigation and from colour photographs and SEM images on the sealant groups (*p* = 0.17).

At evaluation years 2 and 3, 76.1 and 89.6 % of retention code 4 from clinical visible examination were confirmed as a code 4 by the assessment from colour photographs and SEM images, while 2.5 % (2 years) and 4.4 % (3 years) of retention code 4 were scored a retention code 6 (Table 5).

**Table 5:** Clinical retention code 4 against retention scores assessed from images of colour picture and SEM (code) by sealant group for the three sections (mesial-central-distal) of the occlusal surface at evaluation years 2 and 3 combined

Assessment criterium	Year 2				Year 3				Years 2 and 3 together			
	Mesial	Central	Distal	Total	Mesial	Central	Distal	Total	Mesial	Central	Distal	Total
Code 1	6	5	3	14	1	1	0	2	7	6	3	16
Code 2	4	12	13	29	4	2	3	9	8	14	16	38
Code 4	41	45	67	153	42	53	69	164	83	88	136	317
Code 6	0	1	4	5	0	3	5	8	0	4	9	13
Total				201				183				384

### Retention codes 5 and 6 from visible clinical examination at years 2 and 3

The number of surface sections with a retention code 5 or 6 from the clinical examination at evaluation years 2 and 3 was 3 and 21, respectively. Of these, only one code 5 or 6 from the clinical examination was confirmed as a code 6 by the colour photograph and SEM image assessment.

## Discussion

### Research methodology

As no previous study had been performed on this topic, it was not possible to determine the optimum sample size. Despite the fact that the sample number was restricted by the cost of producing the SEM images within the budget, its size of well over 100 teeth per evaluation year, with some having more than one section available for assessment, is considered large enough for answering the research question.

According to protocol, the SEM image was considered the reference standard. However, a number of images were technically unusable for which we did not receive a reason from the technician of the University of Geoscience, who handled the SEM machine on her own. For some we thought that the biofilm was removed from deeper pits and fissures insufficiently, showing foreign bodies. Furthermore, it turned out to be difficult to detect remnants of sealant material with a sufficient level of certainty on a number of SEM images. It was difficult at times to distinguish the surface of an enamel carious lesion from parts of sealant material. For those reasons, the SEM images could not be used solely as the reference standard. As retention of sealant material is adequately assessed from colour photographs than through visible clinical examination [15], we decided to modify the assessment procedure. Tooth sections were assessed from colour photographs immediately followed by the corresponding SEM image. This procedure has the added advantage that any doubtful decision from colour picture could be re-assessed for its correctness. One can argue that, as the combined assessment was not applied on all teeth, the methodology holds a certain level of evaluation bias. That may be correct but considering the low number of unusable SEM images, the strength of using two assessment methods increasing the chance for finding the truth in the majority of cases and the low frequency of remnants observed, we think that through the modified assessment process, an adequate estimate of the real situation was obtained.

### Main findings

The null hypothesis was accepted. No significant difference was found among the sealant groups with respect to the presence of material remnants in pits and fissures from which the sealant had been judged to have completely disappeared according to the visual clinical examination. In fact, the occurrence of remnants at both evaluation years was very low. This finding appears to be different from those described in earlier publications. Describing four cases of ART sealants in position after 8–13 years, Frencken and Wolke [12] observed glass-ionomer-like remnants in pits and fissures in all the cases. The same observation had been reported by Mejàre and Mjör [13] and Torppa-Saarinen and Seppä [14] in vivo and Smith [16] in an in vitro simulation model, which finding could not be observed in the present study. This may suggest that the assumption is unfounded that glass-ionomer remnants left behind in the deeper parts of pits and fissures, making them less deep and easier to clean with brush and toothpaste, is a reason for the absence of a difference in the prevalence of cavitated dentine carious lesions over time between glass-ionomer- and resin-based sealants [4–6]. Reasons that explain this phenomenon may well be related to the level of biofilm control as part of the maintenance programme accompanying the sealants trials referred to. However, this assumption was not a topic of the present study.

Another remarkable finding was the high number of observed retention code 4 scores from assessing resin sealants through visual clinical examination that were scored retention codes 1 and 2 on colour photographs and SEM images at evaluation year 2. This finding was not observed in the other sealant groups. We are unaware regarding factors that may have caused this result. Whatever the reason may be, the result implies that the survival of fully and partially retained resin sealants at evaluation year 2 is likely to be higher than reported [11].

It is concluded that the prevalence of remnants of glass-ionomer sealant in pits and fissures of occlusal surfaces in first permanent molars is not higher than that of glass-carbomer and resin sealants after 2 and 3 years.
